# Sex differences in the radiographic and symptomatic prevalence of knee and hip osteoarthritis

**DOI:** 10.3389/fendo.2024.1445468

**Published:** 2024-10-04

**Authors:** Benjamin G. Faber, Fiona Macrae, Mijin Jung, Benjamin E. Zucker, Rhona A. Beynon, Jonathan H. Tobias

**Affiliations:** ^1^ Musculoskeletal Research Unit, University of Bristol, Bristol, United Kingdom; ^2^ Medical Research Council Integrative Epidemiology Unit, University of Bristol, Bristol, United Kingdom; ^3^ Cardiology Department, Gloucester Royal Hospital, Gloucester, United Kingdom

**Keywords:** osteoarthritis, epidemiology, knee, hip, sex difference

## Abstract

Recognising sex differences in disease prevalence can lead to clues as to its pathogenesis, for example the role of hormonal factors and related influences such as body composition, as well as forming the basis for new treatments. However, if different methods are used to define the disorder it can be difficult to explore differences in prevalence, making it necessary to draw on multiple sources of evidence. This narrative review addresses sex differences in the prevalence of knee and hip osteoarthritis, which are the most common forms of large joint osteoarthritis. Females appear to have a higher prevalence of knee osteoarthritis across a wide range of disease definitions, while findings for the hip vary depending on how the disease is defined. Clinically or symptomatically defined hip osteoarthritis is more common in females, whereas radiographically defined hip osteoarthritis is more common in males. Therefore, understanding sex differences in large joint arthritis requires consideration that osteoarthritis, as defined structurally, more commonly affects females at the knee, whereas the opposite is true at the hip. Furthermore, despite structural changes in hip osteoarthritis being more common in males, symptomatic hip osteoarthritis is more common in females. The basis for these disparities is currently unclear, but may reflect a combination of hormonal, biomechanical and behavioural factors.

## Introduction

Knee and hip osteoarthritis, the most common forms of large joint osteoarthritis, are chronic, disabling, and highly prevalent conditions (1, 2). In the United Kingdom, large joint osteoarthritis has contributed to a significant increase in individuals leaving the workforce, with associated healthcare costs estimated to reach approximately £5 billion per year (3). During the early stages of the disease, primary treatments involve lifestyle interventions, exercise therapy and analgesia (1). In end-stage disease, the definitive treatment is costly joint replacement. Unfortunately, roughly 10-30% of individuals still suffer from chronic pain and loss of function after joint replacement, justifying research into novel treatments (4, 5). One consequence of the increasing prevalence of this condition is the increased demand for joint replacement procedures, placing strain on healthcare services, thus contributing substantially to the societal impact of this disease (6).

Recognising sex differences in disease prevalence can help in understanding pathogenesis and guiding treatment. However, this may be complicated if different methods are used to define the disorder. Previous research has indicated there are large sex differences in knee and hip osteoarthritis, and several comprehensive reviews have explored this topic (7–11). However, these reviews do not address the sex differences in disease prevalence according to disease definitions. Osteoarthritis can be defined clinically, symptomatically, and radiographically for the purpose of epidemiological studies (12). A clinical definition is usually obtained from healthcare records, where an individual has been diagnosed as having osteoarthritis by a healthcare professional. It is generally assumed that these individuals have symptoms, as asymptomatic cases are unlikely to receive a diagnosis. In some studies, osteoarthritis is defined by the concurrent presence of symptoms (such as pain, stiffness, or swelling) and observable radiographic changes (such as joint space narrowing or osteophyte formation) (13). Conversely, other studies adopt a purely radiographic definition (14), relying on established diagnostic criteria (e.g. Kellgren-Lawrence Scoring) (15). Additionally, as total knee and hip replacements are primarily used for the treatment of end stage symptomatic osteoarthritis, these procedures can serve as proxies for disease presence (16). When considering the prevalence of knee and hip osteoarthritis, these definitions should be considered individually as they measure different disease characteristics and are not necessarily interchangeable (17).

Females are reported to have an increased prevalence of osteoarthritis across all joints (18), although conflicting research has indicated that when defined radiographically, hip osteoarthritis is more common in males (19). In addition, prior work has suggested that females often receive fewer healthcare interventions for osteoarthritis, despite their higher prevalence (20, 21). Therefore, this narrative review was initiated to review the current literature regarding sex differences in knee and hip osteoarthritis prevalence according to different disease definitions, to review sex differences in rates of joint replacement, and to consider the possible reasons for the differences found. The focus of this paper will be on knee and hip osteoarthritis, given they are the most common large joints affected by osteoarthritis (2).

## Clinical and symptomatic differences in knee and hip osteoarthritis

### Knee osteoarthritis

Studies have consistently shown that symptomatic and clinical knee osteoarthritis are more prevalent in females than males (**Table 1**). For instance, large scale European studies from Spain and the UK, which are based on healthcare records, reveal a prevalence of clinical knee osteoarthritis ranging from 3.2-3.6% in females, compared to 2.2-2.5% in males (22, 23). In a sizeable meta-analysis of prevalence estimates from Chinese populations, aged predominantly over 40 years old, symptomatic knee osteoarthritis was found to be roughly twice as prevalent in females than males (19.1% females vs 10.9% males). These higher prevalence figures are likely attributed to the advanced ages of the populations studied and to differences in disease definition (24). Prospective cohort studies (25–27) tend to show higher prevalence estimates of symptomatic osteoarthritis compared to population-wide prevalence estimates (22, 23) (**Table 1**). Many diverse smaller studies have also found an increased prevalence of symptomatic knee osteoarthritis in females (20, 21, 28–39). Only one study found the prevalence of clinical knee osteoarthritis to be broadly similar between the sexes (F: 2.4% and M: 2.5%) (40).

**Table 1:** Prevalence of sex-stratified clinical and symptomatic knee osteoarthritis.

Author	Country	Population		Age (years)	OA type	Diagnosis method	OA prevalence (%)		
		Male (%)	Female (%)	Mean (Range/SD)			Total	Male	Female
**Prieto-Alhambra et al., 2014** (22)	Spain	1,529,595 (46.8)	1,737,231 (53.2)	67.3 (SD11.0)	Clinical knee	ICD code	96,222 (2.9)	34,243 (2.2)	61,979 (3.6)
**Swain et al., 2022** (23)	UK	1,690,618		Inclusion ≥20 (NA)	Clinical knee	Coded diagnosis (2017)	NA (2.3)	NA (2.5)	NA (3.2)
**Li et al., 2020** (24)	China	74,908		Inclusion ≥15 (NA)	Symptomatic knee	Assorted	10,937 (14.6)	NA (10.9)	NA (19.1)
**Mork et al., 2012** (28)	Norway	14,766 (49.3)	15,191 (50.7)	43.79 (SD 14.0)	Symptomatic knee	Questionnaire	351 (1.2)	132 (0.9)	219 (1.4)
**Hawker et al., 2000** (20)	Canada	11,930 (42.0)	16,521 (58.0)	M: 69.4 (SD 8.7) F: 70.9 (SD 9.6)	Symptomatic hip *and* knee	WOMAC score ≥39	1,325 (4.7)	332 (2.8)	993 (6.0)
**Tang et al., 2015** (35)	China	8,367 (48.8)	8,761 (51.2)	M: 59.8 (SD 0.2) F: 59.8 (0.2)	Symptomatic knee	Questionnaire (CHARLS)	1,360 (8.1)	478 (5.7)	902 (10.3)
**Park et al., 2017** (34)	Korea	3,830 (42.7)	5,146 (57.3)	Inclusion ≥50 (NA)	Symptomatic knee	Questionnaire	754 (8.4)	260 (6.8)	494 (9.6)
**Welling et al., 2017** (40)	Finland	3,904 (47.3)	4,344 (52.7)	Inclusion ≥46 (NA)	Clinical knee	ICD code	202 (2.5)	98 (2.5)	104 (2.4)
**Zhang et al., 2016** (43)	China	3,609 (50.7)	3,517 (49.4)	43.9 (SD 16.6)	Clinical knee	ACR criteria	983 (13.8)	446 (12.4)	537 (15.3)
**Haq et al., 2005** (36)	Bangladesh	2,582 (50.0)	2,578 (50.0)	Inclusion ≥15 (NA)	Symptomatic knee	Questionnaire and examination	451 (8.7)	190 (7.4)	261 (10.1)
**Plotnikoff et al., 2015** (31)	Canada	1,542 (32.6)	3,189 (67.4)	52.5 (SD 16.5)	Symptomatic knee	Questionnaire	352 (7.4)	146 (9.5)	206 (6.5)
**Ji et al., 2023** (37)	China	1,950 (49.7)	1,974 (50.3)	58.44 (SD 9.2)	Symptomatic knee	ACR criteria	404 (10.3)	126 (6.5)	278 (14.1)
**Tukker et al., 2009** (25)	The Netherlands	1,640 (44.8)	2,024 (55.2)	54.6 (NA)	Symptomatic knee	Questionnaire	547 (14.9)	213 (13.0)	334 (16.5)
**Picavet et al., 2003** (41)	The Netherlands	1,641 (44.8)	2,023 (55.2)	Inclusion ≥25 (NA)	Self-reported knee	Questionnaire	441 (12.0)	166 (10.1)	275 (13.6)
**Grotle et al., 2008** (33)	Norway	1,480 (45.3)	1,786 (54.7)	60.44 (IQR 21)	Symptomatic knee	Questionnaire	233 (7.1)	92 (6.2)	141 (7.9)
**Felson et al., 1987** (39)	USA	587 (41.4)	831 (58.6)	73 (Range 63-94)	Symptomatic knee	X-rays and questionnaire	135 (9.5)	40 (6.8)	95 (11.4)
**Carmona et al., 2001** (30)	Spain	1,014 (46.3)	1,178 (53.7)	Inclusion ≥20 (NA)	Symptomatic knee	ACR criteria	223 (10.2)	58 (5.7)	165 (14.0)
**Macias-Hernandez SI et al., 2020** (32)	Mexico	80 (39.2)	124 (60.8)	57.4 (SD 10.9)	Clinical knee	Questionnaire	40 (19.6)	10 (12.5)	30 (24.2)

ACR, American College of Rheumatology; ICD, International Classification of Diseases; WOMAC, Western Ontario and McMaster Universities Osteoarthritis Index; UK, United Kingdom; USA, United States of America.

### Hip osteoarthritis

In a large prospective study of adults over the age of 40 years old in the UK, hip osteoarthritis, defined by both prolonged hip pain and hospital diagnosis, has been found to be more common in females than males (hip pain lasting ≥3 months: Female 9.8% vs male 6.2% & hospital diagnosed: Female 1.5% vs male 1.1%) (19). Similarly, a comprehensive population study conducted in Spain also revealed a slightly higher prevalence of clinical hip osteoarthritis among females compared to males (1.0% vs 0.8%) (22). In a Korean study examining the prevalence of hip pain, deemed to be osteoarthritic in origin, females exhibited nearly three times higher rates (13.9%) than males (4.6%) (34). A large Canadian study looking at self-reported hip osteoarthritis found females to have a more modest increased risk of symptomatic hip osteoarthritis compared to males (6.6% vs 5.5%) (31). Likewise, several smaller studies also concluded that symptomatic and clinical hip osteoarthritis is more common in females than males (25, 26, 32, 33, 41) (**Table 2**). In contrast, studies that have defined symptomatic hip osteoarthritis using pain with co-existent ipsilateral radiographic osteoarthritis suggest either no sex difference (42) or even a slightly higher prevalence in males than females (27, 43).

**Table 2:** Prevalence of sex-stratified clinical and symptomatic hip osteoarthritis.

Author	Country	Population		Age (years)	OA type	Diagnosis method	OA prevalence (%)		
		Male (%)	Female (%)	Mean (Range/SD)			Total	Male	Female
**Prieto-Alhambra et al., 2014** (22)	Spain	1,529,595 (46.8)	1,737,231 (53.2)	67.3 (SD11.0)	Clinical hip	ICD code	30,349 (0.9)	12,698 (0.8)	17,652 (1.0)
**Swain et al., 2022** (23)	UK	1,690,618		Inclusion ≥20 (NA)	Clinical hip	Coded diagnosis (2017)	NA (1.1)	NA (1.2)	NA (1.7)
**Faber et al., 2022** (19)	UK	19,294 (47.8)	21,046 (52.2)	63.7 (Range 44-82)	Symptomatic hip	Hip pain >3 months Hospital diagnosed	3251 (8.1) 527 (1.3)	1193 (6.2) 220 (1.1)	2058 (9.8) 307 (1.5)
**Mork et al., 2012** (28)	Norway	14,766 (49.3)	15,191 (50.7)	43.79 (SD 14.0)	Symptomatic hip	Questionnaire	322 (1.1)	102 (0.7)	220 (1.4)
**Hawker et al., 2000** (20)	Canada	11,930 (41.9)	16,521 (58.1)	M: 69.4 (SD 8.7) F: 70.9 (SD 9.6)	Symptomatic hip *and* knee	WOMAC score ≥39	1,325 (4.7)	332 (2.8)	993 (6.0)
**Jüni et al., 2010** (21)	UK	12,078 (46.4)	13,968 (53.6)	62.44 (SD 12.0)	Symptomatic hip	New Zealand Score ≥43	256 (1.0)	81 (0.7)	175 (1.3)
**Park et al., 2017** (34)	Korea	3,830 (42.7)	5,146 (57.3)	Inclusion ≥50 (NA)	Symptomatic hip	Questionnaire	891 (9.9)	176 (4.6)	715 (13.9)
**Welling et al., 2017** (40)	Finland	3,904 (47.3)	4,344 (52.7)	Inclusion ≥46 (NA)	Clinical hip	ICD code	40 (0.5)	22 (0.6)	18 (0.4)
**Zhang et al., 2016** (43)	China	3,609 (50.6)	3,517 (49.4)	43.9 (SD 16.6)	Clinical hip	ACR criteria	42 (0.6)	23 (0.6)	19 (0.5)
**Plotnikoff et al., 2015** (31)	Canada	1,542 (32.6)	3,189 (67.4)	52.5 (SD 16.5)	Symptomatic hip	Questionnaire	278 (5.9)	102 (6.6)	176 (5.5)
**Tukker et al., 2009** (25)	The Netherlands	1,640 (44.8)	2,024 (55.2)	54.6 (NA)	Symptomatic hip	Questionnaire	356 (9.7)	107 (6.5)	249 (12.3)
**Picavet et al., 2003** (41)	The Netherlands	1,641 (44.8)	2,023 (55.2)	Inclusion ≥25 (NA)	Self-reported hip	Questionnaire	258 (7.0)	64 (3.9)	194 (9.6)
**Grotle et al., 2008** (33)	Norway	1,480 (45.3)	1,786 (54.7)	60.44 (IQR 21)	Symptomatic hip	Questionnaire	179 (5.5)	68 (4.6)	111 (6.2)
**Jordan et al., 2009** (26)	USA	1,162 (37.9)	1,906 (61.1)	Inclusion ≥45 (NA)	Symptomatic hip	Questionnaire	1,123 (36.6)	370 (31.8)	753 (39.5)
**Kim et al., 2014** (27)	USA	434 (44.4)	544 (55.6)	63.5 (SD 9.0)	Symptomatic hip	X-ray and symptoms	39 (4.0)	23 (5.3)	16 (3.0)
**Macias-Hernandez SI et al., 2020** (32)	Mexico	80 (39.2)	124 (60.8)	57.4 (SD 10.9)	Clinical hip	Questionnaire	37 (18.1)	6 (7.5)	31 (25.0)

ACR, American College of Rheumatology; ICD, International Classification of Diseases; WOMAC, Western Ontario and McMaster Universities Osteoarthritis Index; UK, United Kingdom; USA, United States of America.

## Radiographic osteoarthritis

Radiographic osteoarthritis is a composite diagnosis made on the basis of changes observed in joint imaging. Kellgren and Lawrence described the radiographic features of osteoarthritis as joint space narrowing, osteophytosis, subchondral sclerosis and cyst formation (15).

Studies consistently report higher prevalence figures for radiographic knee osteoarthritis in females over males (32, 34, 38, 44–46) (**Table 3**). In the large prospective Rotterdam Study, radiographic knee osteoarthritis was found to be twice as common in females as males (20.0% vs 9.0%, respectively) (47). A study based in the USA with a high proportion of African Americans found higher prevalence estimates for radiographic knee osteoarthritis, but again a higher prevalence was seen in females (F: 31.0% vs M: 23.7%) (48). The prevalence estimates for radiographic knee osteoarthritis tend to be higher than estimates for symptomatic and clinical osteoarthritis but the sex differences are equivalent (**Tables 1**, **3**), and this is likely due to the structural joint changes (e.g. osteophytes) often being asymptomatic (27). In the aforementioned studies, radiographs (X-rays) were used to define disease, but it is worth noting that high-resolution dual-energy X-ray absorptiometry (DXA) scans are increasingly being used for this purpose (49, 50).

**Table 3:** Prevalence of sex-stratified radiographic knee osteoarthritis.

Author	Country	Population (%)		Age	OA type	Diagnosis method	OA prevalence (%)		
		Male	Female	Mean (Range)			Total	Male	Female
**Park et al., 2017** (34)	Korea	3,830 (46.7)	5,146 (57.3)	Inclusion ≥ 50 (NA)	Radiographic knee	KL grade≥2	1,858 (20.7)	628 (16.4)	1,230 (23.9)
**Hoeven et al., 2012** (47)	Netherland (Rotterdam)	2,372 (42.0)	3,278 (58.0)	M: 67.5 (SD 7.6) F: 68.6 (SD 8.3)	Radiographic knee	KL grade≥2	868 (15.0)	213 (9.0)	655 (20.0)
**Jordan et al., 2007** (48)	USA (Johnston County)	1,162 (37.9)	1,906 (62.1)	Inclusion ≥45 (NA)	Radiographic knee	KL grade≥2	866 (27.8)	275 (23.7)	591 (31.0)
**Muraki et al., 2014** (45)	Japan (ROAD study)	553 (35.5)	1,005 (64.5)	M: 68.1 (SD 10.7) F: 66.5 (SD 11.0)	Radiographic knee	KL grade≥2	769 (49.3)	214 (38.7)	555 (55.2)
**Felson et al., 1995** (38)	USA	313 (36.0)	556 (64.0)	70.8 (SD 5.0) (Range 63-91)	Radiographic knee	KL grade≥2	322 (37.1)	NA (11.1)	NA (18.1)
**Cho et al., 2015** (46)	Korea	298 (42.8)	398 (57.2)	72.0 (SD 5.0) (Range 65-91)	Radiographic knee	KL grade≥2	265 (38.1)	51 (17.1)	214 (53.8)
**Ho-Pham et al., 2014** (44)	Vietnam	170 (25.8)	488 (74.2)	M: 55.1 (SD 15.8) F: 55.9 (SD 12.6) (Range 40-98)	Radiographic knee	KL grade≥2	225 (34.2)	53 (31.2)	172 (35.3)
**Macías-Hernández et al., 2020** (32)	Mexico	80 (39.2)	124 (60.8)	57.4 (SD 10.9) (Range 42-86)	Radiographic knee	KL grade≥2	52 (25.5)	14 (17.5)	38 (30.6)

KL, Kellgren-Lawrence grade; UK, United Kingdom; USA, United States of America.

At the hip, the opposite association is seen in the majority of studies (**Table 4**), with radiographic osteoarthritis appearing to be more common in males as compared to females (27, 34, 46, 51–53). The largest study to date, looking at data from 40,340 individuals over 40 years old in the UK, estimated the prevalence of radiographic osteoarthritis (defined as grade ≥2 on high-resolution DXA scans) to be 8.2% in males compared to 3.5% in females (19). There were three studies that reported a higher prevalence of radiographic hip osteoarthritis in females (26, 32, 47). One of these studies, involving the Johnston County Osteoarthritis Project, was specifically designed to investigate racial differences in osteoarthritis. As a result, Caucasian women over 65 years old were intentionally under-sampled, while African Americans of both sexes were oversampled. This may have led to biased sex-based prevalence estimates, particularly given that osteoarthritis is more common in African Americans (26, 48). Another of these studies, a very small Mexican study, sampled only 204 individuals (32). Whilst radiographic hip osteoarthritis has been shown to be more common in males, this is not always the case for symptomatic radiographic hip osteoarthritis as previously mentioned (27, 42, 43).

**Table 4:** Prevalence of sex-stratified radiographic hip osteoarthritis.

Author	Country	Population (%)		Age	OA type	Diagnosis method	OA prevalence (%)		
		Male	Female	Mean (Range)			Total	Male	Female
**Faber et al., 2022** (19)	UK (UK Biobank)	19,294 (47.8)	21,046 (52.2)	M: 64.4 (Range 44-81) F: 63.0 (Range 45-82)	Radiographic hip	KL grade≥2	3,017 (7.5)	2,086 (10.8)	931 (4.4)
**Park et al., 2017** (34)	Korea	3,830 (46.7)	5,146 (57.3)	Inclusion ≥ 50 (NA)	Radiographic hip	KL grade≥2	57 (0.6)	42 (1.1)	15 (0.3)
**Hoeven et al., 2012** (47)	Netherland (Rotterdam)	2,372 (42.0)	3,278 (58.0)	M: 67.5 (SD 7.6) F: 68.6 (SD 8.3)	Radiographic hip	KL grade≥2	348 (6.0)	119 (5.0)	229 (7.0)
**Jordan et al., 2009** (26)	USA (Johnston County)	1,162 (37.9)	1,906 (62.1)	Inclusion ≥45 (NA)	Radiographic hip	KL grade≥2	857 (27.6)	295 (25.4)	562 (29.5)
**Iidaka et al., 2016** (52)	Japan (ROAD study)	1,043 (35.1)	1,932 (64.9)	M: 71.0 (SD 10.7) F: 69.8 (SD 11.3) (Range 23-94)	Radiographic hip	KL grade≥2	467 (15.7)	190 (18.2)	277 (14.3)
**Tepper and Hochberg, 1993** (53)	US (NHANES-I)	2,358		NA (Range 55-74)	Radiographic hip	KL grade≥2	73 (3.1)	NA (3.2)	NA (3.0)
**Kim et al., 2014** (27)	USA	434 (44.4)	544 (55.6)	63.5 (SD 9.0) (Range 51-92)	Radiographic hip	KL grade≥2	181 (18.5)	107 (24.7)	74 (13.6)
**Hirsch et al., 1998** (51)	US (Pima Indians)	294 (38.9)	461 (61.1)	M: 58.1 (SD 9.9) F: 58.4 (SD 9.1) (Range 45-93)	Radiographic hip	KL grade≥2	27 (3.6)	14 (4.8)	13 (2.8)
**Cho et al., 2015** (46)	Korea	298 (42.8)	398 (57.2)	72.0 (SD 5.0) (Range 65-91)	Radiographic hip	KL grade≥2	15 (2.2)	8 (2.7)	7 (1.8)
**Macías-Hernández et al., 2020** (32)	Mexico	80 (39.2)	124 (60.8)	57.4 (SD 10.9) (Range 42-86)	Radiographic hip	KL grade≥2	54 (26.5)	15 (18.7)	39 (31.4)

Although it is known that radiographic features do not perfectly correlate with symptoms (17), they are strongly associated in both sexes (19, 49, 54, 55). However, it has been shown that females tend to experience more severe pain than males with equivalent radiographic changes (56). In addition, at the hip, females show a stronger association between radiographic changes and both symptoms and total hip replacement (19). Any tendency for females to experience worse pain for a given degree of structural change could explain their higher prevalence of clinical/symptomatic as opposed to radiographic hip osteoarthritis. Such differences might also be expected to translate into sex differences in the rate of interventions such as joint replacement.

## Sex differences in rates of hip and knee joint replacement

Based on the data presented, it would be reasonable to anticipate that females would constitute a larger proportion of total joint replacements given their higher disease burden. The National Joint Registry collates information on all joint replacements conducted in England and Wales from both public and private healthcare providers. In 2022, 100,095 total knee replacements were performed, with a higher number of cases in females (n=54,731- 55%) than male (n=45,364 – 45%), despite similar mean ages seen between the groups (females: 70.0 vs males: 69.7 years) (16). Regarding hip replacements, during the same time period, 95,880 primary hip replacements were conducted in England and Wales (Female 60,687 [63.3%] vs Male 31,308 [36.7%], mean age F: 70.22 vs M: 67.8 years) (57). Data from the American Joint Replacement Registry suggests, between 2012-2022, roughly 62% of primary total knee replacements and 57% of primary total hip replacements were done in females (58). These data show that in both the UK and USA, females receive a greater proportion of joint replacements. The largest observational studies already presented (**Tables 1**, **2**) suggest that symptomatic knee osteoarthritis is roughly 30% more common in females (22–24), and symptomatic hip osteoarthritis is 40% more common in females (19, 22, 23). Therefore, the allocation of joint replacements reported in the UK and USA would appear to be in line with other measures reflecting sex-differences in symptomatic knee and hip osteoarthritis prevalence.

As well as sex differences in the rate of joint replacements, it has been shown that females often have worse symptoms prior to joint replacement than males (59). This is highlighted by data that showed females in the twelve months before surgery were more likely to use analgesia and seek healthcare than males (60). Qualitative studies suggest that females might delay joint replacement for several reasons: they often exhibit greater apprehension about surgery (61), tend to have more questions prior to surgery (62) and prioritise avoiding surgery more than males do (63). Clinicians caring for patients with large joint osteoarthritis should consider these differences in patient-level factors, particularly regarding decisions about proceeding to surgery. In addition, there is evidence to suggest that clinician-related factors can lead to sex-based discrepancies in care. Studies have shown that females are less likely to receive a referral to an orthopaedic surgeon when presenting with the same symptom levels as males (64). In the case of hip osteoarthritis, this may be attributed to the greater prevalence of structural disease in males, leading to surgeons prioritising them for treatment.

Following joint replacement surgery, it has been found that females report worse outcomes in pain and function (59). This might be because they are at a more advanced disease stage when their surgery takes place (11). Alternatively, it might be due to sex differences in the experience of musculoskeletal pain (8). We will now consider the potential aetiological reasons for the observed sex differences described so far.

## Potential aetiologic mechanisms

Studies comparing sex differences in the prevalence of knee and hip osteoarthritis show a greater prevalence of symptomatic and clinically defined osteoarthritis in females. A similar female predominance is also seen in relation to rates of joint replacement. Consistent with these findings, when examining sex differences in structural changes of osteoarthritis, as reflected by radiographic osteoarthritis, females show a higher prevalence of radiographic knee osteoarthritis. On the other hand, radiographic hip osteoarthritis is more prevalent in males. These somewhat discrepant findings could be explained by the existence of both sex-based effects on structure which are joint specific, and more generalised sex differences in pain perception. Understanding the underlying mechanism of these differences could shed light on the pathogenesis of osteoarthritis.

### Hormonal

The influence of sex hormones on large joint osteoarthritis has been extensively studied (8). Most studies showing sex differences in the prevalence of knee and hip osteoarthritis are focused on older populations (**Tables 1**, **2**), where the females are likely to be post-menopausal (65). There are established links between menopause and increased rates of hip and knee replacement (66). Indeed, one study found that females whose age at menopause was 50–54 years had a hazard ratio of 0.89 (95% CI 0.84-0.94) for undergoing total knee replacement when compared to females whose age at menopause was 40 years or younger (66). Genetic factors have also been implicated in the association between sex steroid levels and osteoarthritis. The Genetics of Osteoarthritis consortium found 3 genetic risk loci that were female specific. Two of these were associated with total hip replacement and one was associated with osteoarthritis at all sites (67). One of these loci (*FANCL*) was associated with early menopause.

In addition to the association between endogenous sex steroids and osteoarthritis, attempts have been made to elucidate whether exogenous sex steroids affect osteoarthritis. Preclinical studies indicate that selective oestrogen receptor modulators (SERMs) treatment has consistently positive effects on osteoarthritis, especially for postmenopausal patients with early-stage osteoarthritis (68). Despite these promising data, the use of exogenous sex hormones has not yet shown efficacy at a clinical level for osteoarthritis, although trials investigating the use of exogenous oestrogen for knee osteoarthritis in females are ongoing (69).

In terms of the mechanisms by which sex hormones might influence osteoarthritis, oestrogen has been found to protect against articular cartilage and subchondral bone degradation, likely through the sex hormone receptors expressed by osteoblasts, osteoclasts and chondrocytes (9, 70, 71). However, since any such chondroprotective effect would be expected to be generalised, it is difficult to understand how this might underlie the higher risk of structural deterioration of knee joints in females, but not hip joints. On the other hand, sex hormones might contribute to sex differences in pain perception.

### Sex differences in pain perception and related behaviours

Pain is a subjective experience that encompasses sensory, emotional and cognitive components (72). Important differences are thought to exist in how pain is felt between males and females, which may help to explain why symptomatic hip osteoarthritis is more common in females, despite radiographic hip osteoarthritis being more prevalent in males. It has been shown in animal models that both central and peripheral neuronal signalling of pain is highly sexually dimorphic due to greater peripheral nociceptor plasticity (73), altered dopaminergic signally within the spinal column (74), and reduced downregulation of pain within the midbrain (periaqueductal gray) (72) in females. In human studies, females have been shown to have higher activation of their prefrontal cortex in response to painful stimuli and this is thought to lead to an increased perception of pain (75, 76). High concentrations of oestradiol have been found to carry an anti-nociceptive effect, whereas it may be pronociceptive at lower concentrations (8). In addition, oestrogens have been found to decrease proinflammatory cytokine production in the synovial membrane (77). Although the precise causal mechanisms for this are unknown, females have been shown to have a tendency to upregulate pain pathways through increased transcriptional activity of pain related genes (72). Taken together, these mechanisms likely contribute to the increased burden of symptomatic osteoarthritis in females.

Another potential reason why clinically defined osteoarthritis is more common in females is that they have been shown to seek more healthcare input from primary care, which could lead to more recorded diagnoses (78). Recent studies have looked at dispositional traits and their association with knee osteoarthritis. Dispositional traits, which are neurobiologically based, can be divided into groups comprising ‘protective’ or ‘vulnerable’ dispositional traits. Whilst there is no association between sex and dispositional traits, a strong association has been found between ‘vulnerable’ dispositional traits and pain threshold (79). Therefore, the observed sex differences in healthcare utilisation and clinical knee and hip osteoarthritis likely reflect a combination of sex-related differences in disease course and sociocultural differences in healthcare access (80).

### Body composition

Obesity is a risk factor for the development of knee and hip osteoarthritis, particularly knee osteoarthritis, and differences in relative weight seen between the sexes might explain some of the variance in disease prevalence. For example, the association between knee osteoarthritis and obesity has frequently been found to be stronger in females (81). The reasons for this are likely multifactorial. High body mass results in excess joint loading and this phenomenon might be exacerbated by sex differences in fat distribution (82). Moreover, the problem of increased load is further compounded in females due to their relatively reduced cartilage volume (83). This combination may contribute to the increased rate of cartilage loss observed in females compared to males (9). In addition, obesity is a key component of metabolic syndrome, which further includes hypertension, dyslipidaemia, hyperglycaemia, and insulin resistance that are also thought to independently contribute to increased osteoarthritis risk (84). Metabolic syndrome has been shown to be more common in females so this may contribute to the increased prevalence of osteoarthritis in females (85, 86).

Females have decreased lean mass, a proxy for muscle strength, relative to men and increased fat mass (87). This may confer an additional sex-related risk factor for osteoarthritis, as low skeletal muscle mass has been found to be associated with knee osteoarthritis (88). Mechanistic pathways by which lower-limb skeletal muscle effects a reduction in knee osteoarthritis have been proposed (89). Skeletal muscle, known to be more abundant in males, has been suggested to mediate an anti-inflammatory effect and increase resistance of chondrocytes to cytokine induced cartilage damage (90). Furthermore, skeletal muscle may exert a direct positive effect on cartilage by enhancing expression of the dominant (type II) and stabilising (type IX) collagen in cartilage (91).

Sex differences in obesity and body composition are unlikely to account for the higher prevalence of radiographic hip osteoarthritis in males. Interestingly, the association between weight and osteoarthritis at the hip is relatively weak compared with the knee (92). Whilst height is a strong risk factor for radiographic osteoarthritis at the hip (93, 94). Therefore, it is probable that sex differences in biomechanics play a greater role at the hip than obesity, which we will now discuss.

### Biomechanical

Sex-related biomechanical differences at the hip may contribute to the increased prevalence of radiographic osteoarthritis in men (27). This could be partly explained by the increased prevalence of cam morphology of the hip (a bulging aspherical femoral head) in males compared to females (95), given the strong association between cam and hip osteoarthritis (96). In addition, larger lesser trochanters have been implicated as a risk factor for hip osteoarthritis (97). As males have larger lesser trochanters, this risk factor may contribute to the increased rate of radiographic hip osteoarthritis observed in men (98). Though the mechanism by which this risk is conferred is unclear, it has been suggested that aberrant joint forces conferred by the iliopsoas through the lesser trochanter may contribute to the development of hip osteoarthritis (97).

Acetabular dysplasia is a reported risk factor for the development of hip osteoarthritis, as it increases joint load and accelerates cartilage wear (99). Acetabular dysplasia has been reported to have a general prevalence of 3.4%, making it a common risk factor for the development of osteoarthritis (100). While some studies report that acetabular dysplasia is more common in females (99), this finding lacks consistency, with other studies suggesting that the sex-related differences in acetabular dysplasia are unlikely to be of clinical significance (100).

**Figure 1:**
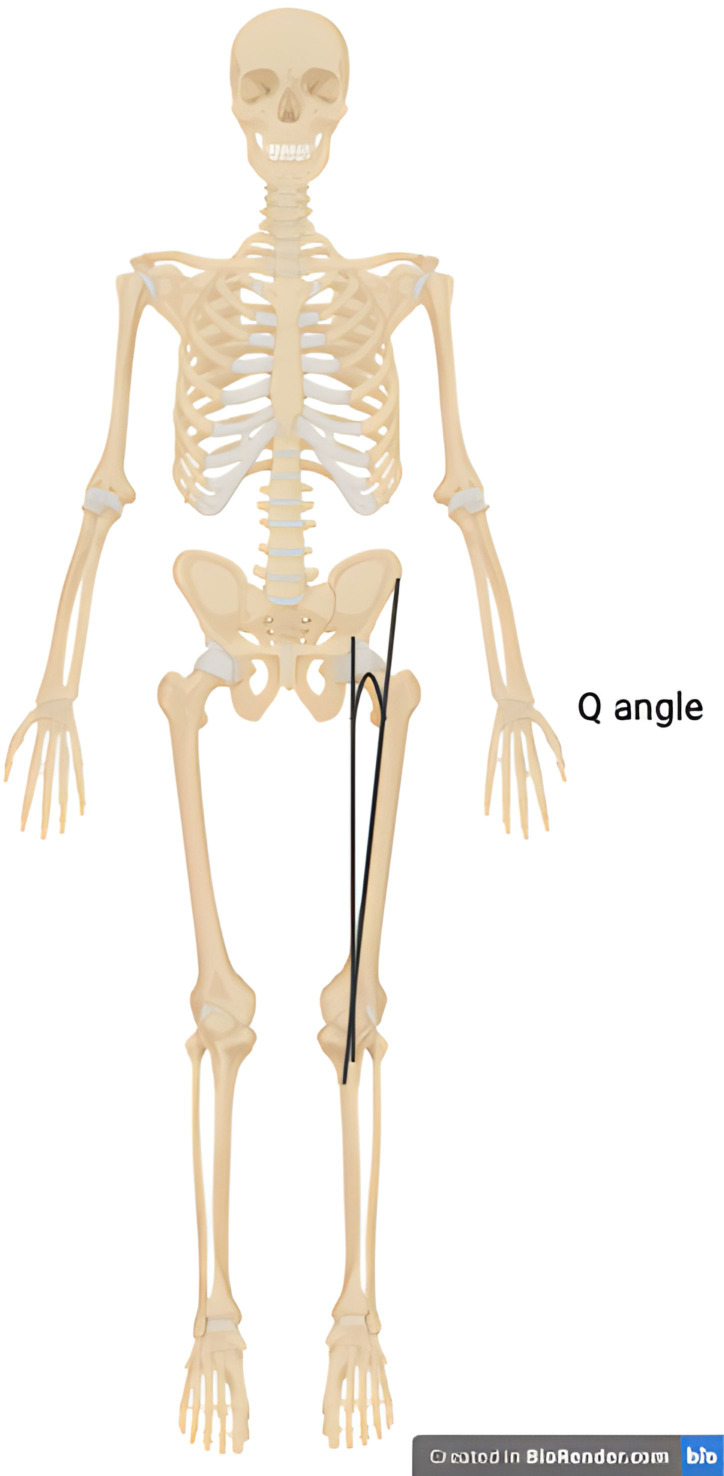
Illustration of the Q angle. The Q angle is defined as the angle between a line that passes through the centre of the patella and the tibial tubercle and a line that pass through the centre of the patella and the anterior superior iliac spine.

Sex-related biomechanical risk factors for both medial and lateral tibiofemoral osteoarthritis have been proposed (101, 102). Whilst varus (bow-legged) deformity of the knee is significantly more common in knee osteoarthritis overall, the incidence of valgus (knock-knee) deformity in females with osteoarthritis is elevated relative to men with osteoarthritis (103). The Q angle is the angle of pull on the patella and is defined by the angle between the centre of the patella and the tibial tubercle and a line between the centre of the patella and the anterior superior iliac spine (**Figure 1**). As females have wider pelvises, this angle is greater and may result in load shifting to the lateral compartment and increased risk of lateral tibiofemoral osteoarthritis (101). In addition, a magnetic resonance imaging-based evaluation of knees throughout the stages of osteoarthritis found that females have increased medial tibiofemoral contact area and a reduced congruity index at all stages of osteoarthritis. This joint configuration may predispose females to the development of both symptomatic and radiographic knee osteoarthritis (102).

## Conclusion

Our literature review shows that knee and hip osteoarthritis is more common in females than males when defined clinically or symptomatically, irrespective of country or ethnicity. Similar female predominance is also seen in relation to rates of joint replacement. When large joint osteoarthritis is defined radiographically, knee osteoarthritis remains more common in females than males, while the opposite holds true for the hip, where male disease predominates. The underlying reasons for these sex differences in knee and hip osteoarthritis prevalence are currently unclear, yet factors such as differing hormone levels, pain perception, body composition and pelvic architecture between the sexes may contribute. Developing a better understanding of the biological mechanisms that lead to the observed sex differences in large joint osteoarthritis could offer opportunities to develop therapies that could benefit both sexes, highlighting the need for further investigation.
